# Nicotinic filtering of sensory processing in auditory cortex

**DOI:** 10.3389/fnbeh.2012.00044

**Published:** 2012-07-19

**Authors:** Raju Metherate, Irakli Intskirveli, Hideki D. Kawai

**Affiliations:** ^1^Department of Neurobiology and Behavior, Center for Hearing Research, University of California, Irvine, IrvineCA, USA; ^2^Faculty of Engineering, Department of Bioinformatics, Soka UniversityHachiouji, Tokyo, Japan

**Keywords:** nicotine, acetylcholine, mouse, rat, attention

## Abstract

Although it has been known for decades that the drug nicotine can improve cognitive function, the nature of its effects and the underlying mechanisms are not well understood. Nicotine activates nicotinic acetylcholine (ACh) receptors (nAChRs) that normally are activated by endogenous ACh, presumably “hijacking” the cholinergic contribution to multiple cognitive functions, notably attention. Thus, studying nicotine's effects helps to better understand a commonly used drug as well as functions of nAChRs. Moreover, nicotinic agonists are being developed to treat a variety of disorders that involve attention-related or age-related cognitive dysfunction. Studies have shown that nicotine can enhance processing of attended stimuli and/or reduce processing of distracters; that is, nicotine enhances attentional filtering. To examine potential mechanisms within sensory cortex that may contribute to cognitive functions, here we describe nicotinic actions in primary auditory cortex, where well-characterized neural “filters”—frequency receptive fields—can be exploited to examine nicotinic regulation of cortical processing. Using tone-evoked current-source density (CSD) profiles, we show that nicotine produces complex, layer-dependent effects on spectral and temporal processing that, broadly speaking, enhance responses to characteristic frequency (optimal) stimuli while simultaneously suppressing responses to spectrally distant stimuli. That is, nicotine appears to narrow receptive fields and enhances processing within the narrowed receptive field. Since basic cortical circuitry and nAChR distributions are similar across neocortex, these findings may generalize to neural processing in other sensory regions, and to non-sensory regions where afferent inputs are more difficult to manipulate experimentally. Similar effects across sensory and non-sensory cortical circuits could contribute to nicotinic enhancement of cognitive functions.

## Introduction: nicotinic enhancement of cognitive function

It has been known for decades that nicotine can enhance cognitive function (Terry et al., 1996; Levin et al., 2006; Evans and Drobes, 2009; Sarter et al., 2009). In dozens of studies of animal and human behavior, performance on a variety of tasks is improved by systemic administration of nicotine or agonists specific for nicotinic acetylcholine (ACh) receptors (nAChR) subtypes, and impaired by nicotinic antagonists or disease-induced loss of nAChRs. The α4β2^*^ nAChR subtype that contains α4 and β2 subunits (asterisk indicates presence of additional subunits) is thought to be especially important for regulating cognitive function, but α7 nAChRs that contain only α7 subunits also may be involved. Although it is not understood precisely which cognitive functions are affected nor the underlying mechanisms, nicotine is presumed to “hijack” the endogenous cholinergic contribution to cognitive functions, especially attention, learning, and memory (Kassel, 1997; Levin and Simon, 1998; Dani and De Biasi, 2001; Miwa et al., 2011).

Studying nicotinic actions therefore is useful not only for understanding the effects of a commonly used drug, but also for understanding the role of endogenous ACh and nAChRs in cognitive function. Moreover, there is intense interest in developing selective nicotinic agents that bind either to α7 or α4β2^*^ nAChRs for therapeutic uses, e.g., for attention disorders in adolescents and adults, cognitive decline in Alzheimer's Disease and other dementias, and other disorders such as schizophrenia (Levin et al., 2006; Taly et al., 2009).

There is some uncertainty over which cognitive functions in humans are enhanced by nicotine. Studies of smokers can be complicated by a common, though not universal, requirement that subjects abstain from smoking for some period of time prior to testing. “Pre-nicotine” measurements, therefore, may be more accurately described as reflecting withdrawal from nicotine rather than baseline measures, and nicotine's effects likely reflect relief from withdrawal. However, a recent meta-analysis of 41 nicotine studies on non-smokers or smokers who were not deprived prior to testing indicates consistent enhancement of motor function, attention, and memory (Heishman et al., 2010). The authors explicitly note that no studies were found that measured sensory abilities, and also note that findings of cognitive improvement sometimes were small in magnitude and contradicted by other results. It is worth noting that analyses of nicotine's effects on human cognition can produce some ambiguity that is puzzling, especially given clear findings of nicotinic enhancement of cognitive ability in animal studies (above). It has been suggested that task difficulty must be controlled carefully, since nicotinic effects may become more obvious as the task becomes more difficult (see below) (Evans and Drobes, 2009; St. Peters et al., 2011).

The abundance of research on nicotine's cognitive effects is matched by studies at the cellular level, with considerable information available on the molecular biology, cellular physiology, and brain distribution of nAChRs (Jones et al., 1999; Leonard and Bertrand, 2001; Dani and Bertrand, 2007; Miwa et al., 2011). While work remains to be done at every level, mechanistic links from the cellular to behavioral levels are especially unclear, and information on how nAChRs function within specific cortical circuits is needed to understand nicotinic functions in cognition. A better understanding of how nicotine's cellular actions affect neural circuits also may provide insight into what kinds of cognitive functions are enhanced, and better inform the use of nicotinic agents as treatments.

Sensory systems serve as useful models at the circuit/systems level because it is possible to activate neural circuits physiologically using sensory stimuli, thus enabling studies that integrate information across levels from molecular to systems to behavior. Recent work from our laboratory has focused on understanding regulation of neural systems in rodent auditory cortex, for example examining the role of different neural circuits in establishing sensory receptive fields (Kaur et al., 2004, 2005; Metherate, 2011). Because thalamocortical and long-distance intracortical pathways may convey information about the center and edge, respectively, of receptive fields in auditory cortex, differential regulation of these circuits by nAChRs could have important consequences for sensory-cognitive function. We explore this issue in the following sections.

## Nicotinic ACh receptors and sensory “attentional narrowing”: behavioral and physiological evidence

There is abundant evidence that systemic administration of nicotine enhances sensory-evoked responses recorded within or near auditory, visual, or somatosensory cortex in animals and non-smoking humans (Guha and Pradhan, 1976; Bringmann, 1994; Harkrider and Champlin, 2001; Penschuck et al., 2002; Oldford and Castro-Alamancos, 2003; Metherate, 2004; Liang et al., 2006). Although nicotine can affect cortical sensory processing via nAChRs located subcortically throughout each sensory system (e.g., Morley and Happe, 2000), or by activating diffuse neuromodulatory systems that themselves regulate cortical responses (Lewandowski et al., 1993; Azam et al., 2002, 2003; Hasselmo and Sarter, 2011), the effects of systemic nicotine on sensory-evoked cortical responses are reduced by direct intracortical injection of nAChR antagonists (Parkinson et al., 1988; Liang et al., 2006; Kawai et al., 2007, 2011; Intskirveli and Metherate, 2012), indicating direct actions within the cortex or on thalamocortical afferent inputs. Effective nAChR antagonists include mecamylamine and dihydro-β-erythroidine (DHβE), but not methyllycaconitine (MLA), implying a role for α4β2^*^ but not α7 nAChRs. Importantly, in some cases reduction of evoked responses occurred upon delivery of antagonist alone, i.e., in the absence of exogenous nicotine, implying that release of endogenous ACh acts at nAChRs to maintain sensory responsiveness.

**Figure 1:**
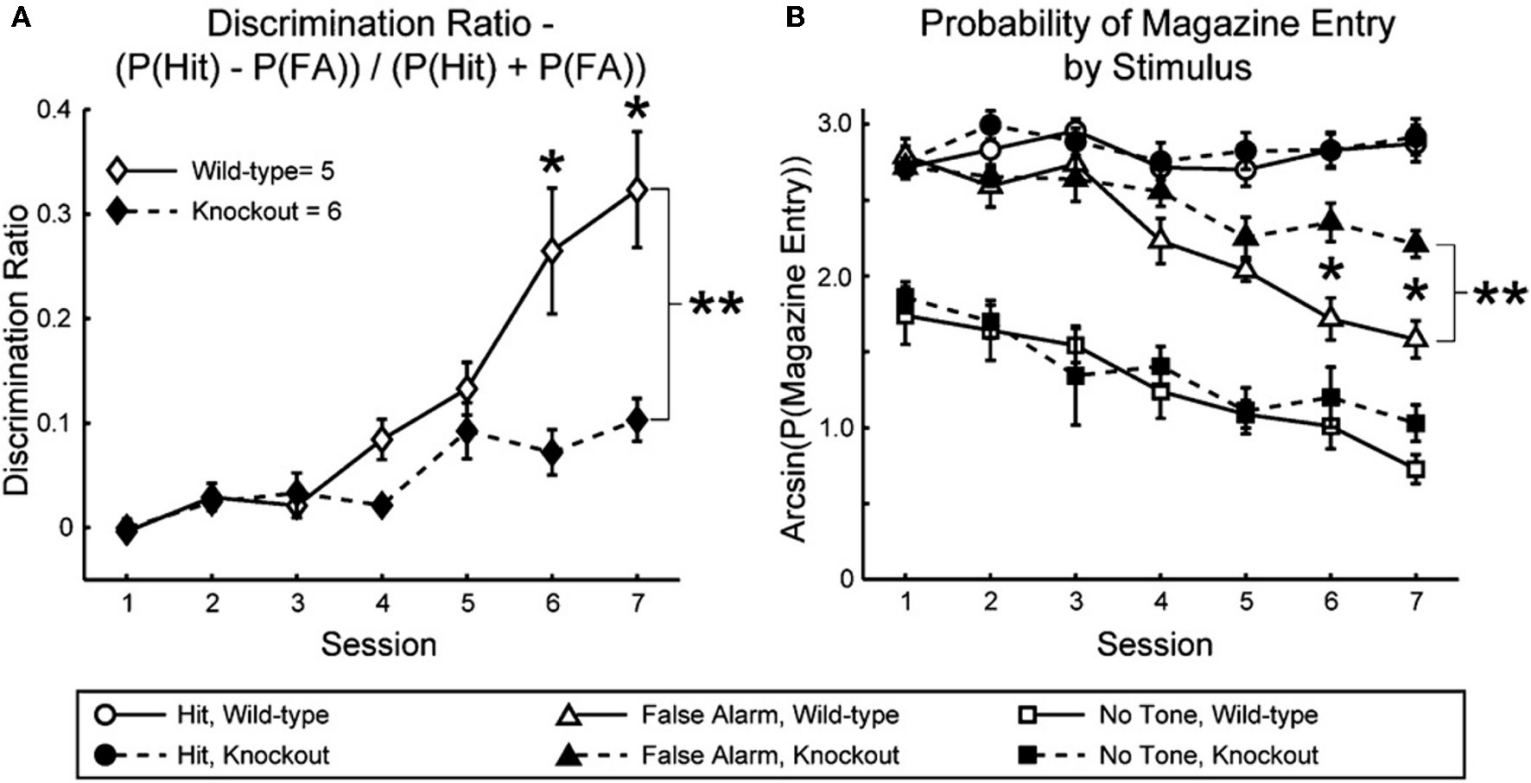
**Impaired sensory discrimination in mice lacking the β2 nAChR subunit. (A)** Discrimination ratio indicating auditory discrimination between rewarded and unrewarded tones in β2 wild-type and knockout mice. **(B)** Arcsine-transformed probability of magazine entry (attempted reward retrieval) in β2 wild-type and knockout mice following the rewarded tone (“Hit”), unrewarded tone (“False Alarm”), and after a nose-poke response when no stimulus was presented (“No Tone”). Stimuli were 12 kHz or 15 kHz tones. ^**^p < 0.05, significant main effect of genotype by repeated measures ANOVA. Reproduced with permission from Horst et al., 2012.

Nicotinic enhancement of sensory responses is linked to enhancement of cognitive function. Nicotine and other nAChR agonists enhance performance on tasks that involve attention to behaviorally relevant stimuli (Warburton, 1992; Evans and Drobes, 2009; Sarter et al., 2009), and while the demonstrated effects of nicotine in studies of attention likely involve non-sensory regions (e.g., prefrontal cortex), effects of nicotine in sensory studies (referenced above) may reflect mechanisms intended for attention-related enhancement of sensory responses. Consistent with this notion, behavioral discrimination of pure-tone stimuli is dramatically impaired in mice lacking β2^*^ nAChRs (Figure 1), whereas the same animals are not impaired in a task that involves similar behaviors but without sensory cues (Horst et al., 2012). It is possible that attention to behaviorally relevant sensory cues depends on nAChR-mediated enhancement of cue-evoked responses, and that similar effects result from the administration of exogenous nicotine.

**Figure 2:**
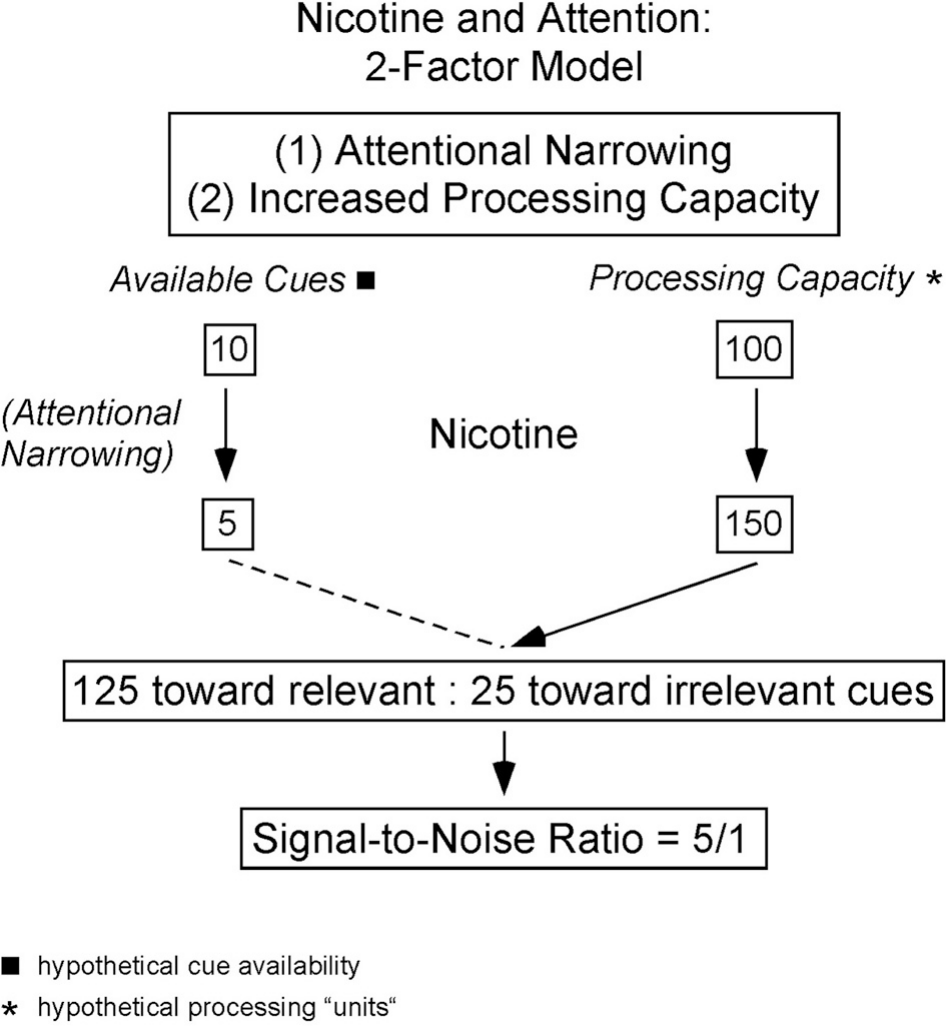
**Hypothetical model in which nicotine's effects on attention are the result of reduced cue utilization via attentional narrowing (left side) and increased processing capacity (right side).** The dual processes result in an enhanced signal-to-noise ratio as more relevant cues are processed than in the absence of nicotine. Figure used with permission and redrawn from Kassel, 1997.

In addition to increasing attention to relevant sensory stimuli, nicotine has been reported to reduce the effectiveness of irrelevant distractors. Several authors have proposed that nicotine improves “attentional narrowing” or stimulus filtering to help focus attention on relevant stimuli (Figure 2). Friedman and colleagues (1974) observed faster habituation to an acoustic cue after smoking, and suggested that nicotine enhances a “stimulus barrier” that suppresses distracting sensory cues (Friedman et al., 1974). Similar hypotheses posit that nicotine enhances attention to relevant stimuli, or suppresses the effect of distractors, or both (Kassel, 1997; Knott et al., 2009). Kassel's formulation of the stimulus-filter hypothesis is shown in Figure 2, which schematically illustrates two components to nicotine's effects on attention: attentional narrowing as well as increased processing capacity for attended stimuli.

The hypothesis in Figure 2 implies two separable functions that should be detectable physiologically: (i) enhanced processing of attended stimuli, and (ii) reduced processing of distractors. Depending on the underlying mechanism the two processes may be independent, or alternatively, interdependent so that increased processing of attended stimuli necessarily is associated with reduced processing of distractors (e.g., attentional resources are limited). As mentioned above, the frequently observed enhancement of sensory responses by nicotine could reflect mechanisms intended to enhance processing of attended stimuli. Physiological evidence for reduced processing of distractors also exists, as nicotine reduces some evoked-potential measures of distractor-evoked responses (Knott et al., 2009).

**Figure 3:**
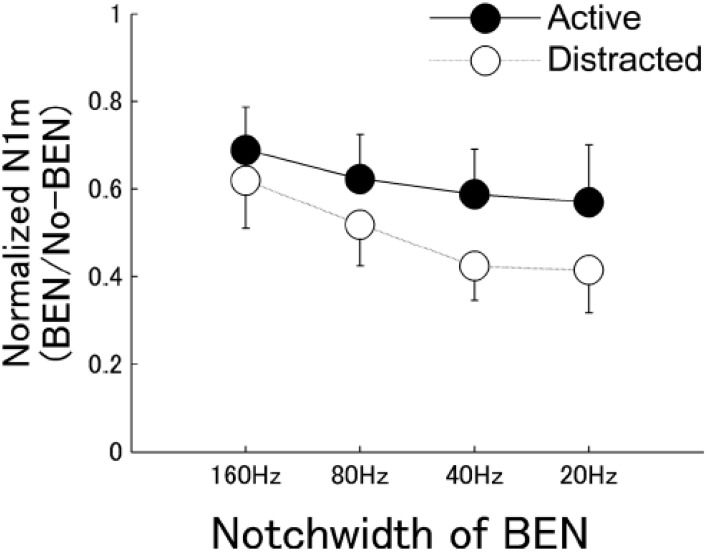
**Normalized magntoencephalography (N1m) source strengths in human cortex.** Graph shows group means of the normalized N1m source strengths for each BEN condition. Filled circles indicate response during active listening; open circles indicate response during distracted listening. BEN, band-eliminated noise. Reproduced with permission from Okamoto et al., 2007.

If the effects of nicotine on sensory-evoked responses reflect hijacked attention mechanisms, then similar effects should be observed in studies of attention *per se*. Indeed, attention can transiently enhance physiological responses in sensory cortex to attended stimuli, reduce responses to unattended stimuli, or both (Miller et al., 1972; Moran and Desimone, 1985; Fritz et al., 2003). In an intriguing study of human sensory processing, Pantev and colleagues used magnetoencephalography to infer a narrowing of frequency tuning throughout auditory cortex during attention to a pure-tone target stimulus (Okamoto et al., 2007). The authors used sustained band-eliminated, or “notched,” noise (BENs) to habituate much of auditory cortex, and then presented a pure-tone target (spectrally centered in the notch) to elicit responses in neurons not fatigued by the BEN. The authors found that attention enhanced target-evoked responses to a greater degree when the notch width was narrower than when it was wider (Figure 3). The implication is that attention-induced narrowing of tuning would leave more neurons unaffected by the sustained BEN and therefore available to respond to the target stimulus. The authors conclude that attention simultaneously increases gain and sharpens tuning at the population level in auditory cortex. Interestingly, this scheme resembles the proposed effects of nicotine (Figure 2).

A variety of studies suggest that effects of nicotine to enhance and filter sensory processing are more prominent under conditions of higher attentional demand. For example, as mentioned above, Knott et al. (2009) showed that nicotine decreased the amplitude of responses evoked by distractors; however, behavioral performance did not improve and the authors suggest that the task was not sufficiently difficult for the neural effect to be reflected in performance. Similarly, a novel and complementary approach to the same issue has shown that a common genetic variant thought to alter α4^*^ nAChR function results in increased attentional performance, but only for tasks with higher processing demands, i.e., with greater number and complexity of distractors (Espeseth et al., 2010). Finally, the results of Horst et al. (Figure 1) that are consistent with a nAChR contribution to sensory function (since performance on a non-sensory task was not impaired), also support a role for nAChRs in attentional narrowing if sharpening of receptive fields is required for discrimination between similar frequencies. Specifically, the authors observed a greater number of false positive responses in mice lacking β2^*^ nAChRs (Figure 1B), but no fewer “hits,” suggesting an inability to discriminate among similar stimuli rather than an inability to detect them. If so, then deficits would be expected for difficult discriminations, i.e., for stimuli that are spectrally close, but not for stimuli that are spectrally distant, a prediction that could be tested in future studies.

Despite the emphasis of this review on mechanisms involving sensory cortex, especially in the sections to follow, it is important to note that nicotinic filtering undoubtedly involves brain regions outside of sensory cortex, e.g., prefrontal cortex and other regions mediating “top-down” control of sensory processing (Knott et al., 2009; Fritz et al., 2010; Hasselmo and Sarter, 2011; St. Peters et al., 2011). However, the purpose of this review is to explore the proposal that nicotinic attentional mechanisms may regulate receptive fields within sensory cortex. We focus on narrowing of receptive field tuning because of the effects of systemic nicotine (below), but it should be noted that attention has been associated with a variety of receptive field changes (Seriès et al., 2004; Reynolds and Heeger, 2009). Nicotinic narrowing of receptive fields, if verified, would form only part of a wider network of mechanisms and brain regions involved in attention. Nonetheless, overall, nicotinic modulation of sensory filtering is a critical control point for regulation of information processing.

## Nicotinic regulation of response selectivity in sensory cortex

Sensory systems have well-characterized, information processing “filters”—sensory receptive fields—that likely contribute to perceptual filters and can be exploited to examine mechanisms of information processing in humans and animal models (e.g., Figure 3). If nicotine's effect on attention involves altered response selectivity in sensory cortex, such effects would be evidenced as changes to receptive fields. Nicotinic regulation of receptive fields may come about via direct activation of nAChRs within sensory cortex and subcortical sensory relays, via top-down regulation by higher cortical regions, or via other mechanisms, but in all cases would have consequences for subsequent information processing.

Many studies have demonstrated nicotinic enhancement of cortical responses to sensory stimuli (see previous section), but fewer have examined regulation of response selectivity. In primate visual cortex, local application of ACh enhanced responses to the central portion of the receptive field while reducing responses to the periphery, leading to a change in length tuning preference towards shorter bar lengths (Roberts et al., 2005). In another visual cortex study, local application of nicotine enhanced response amplitudes and lowered response thresholds to visual contrast stimuli, but only in the thalamocortical input region, layer 4 c (Disney et al., 2007). Outside of the input layer, nicotine tended to have little effect, or a suppressive effect. In rodent somatosensory (“barrel”) cortex, cortical application of nicotine enhanced responses to sensory (whisker) stimulation (Penschuck et al., 2002; Oldford and Castro-Alamancos, 2003), but did not affect intracortical pathways activated by nearby electrical stimulation (Oldford and Castro-Alamancos, 2003). Finally, surface application of an agonist selective for α4^*^ nAChRs exerted mostly suppressive effects on whisker-evoked responses in upper layers (Brown et al., 2012). In general these findings all show or imply that nAChRs regulate response selectivity, but the diversity of findings likely results, in part, from differences in agonist used, method of drug application, type of sensory stimulation, laminar location and type of responding neurons, and other technical differences. It will be important to control for the cortical circuitry being tested, as well as methodological differences, since similar nicotinic actions may contribute differently to different cortical circuits and functions.

**Figure 4:**
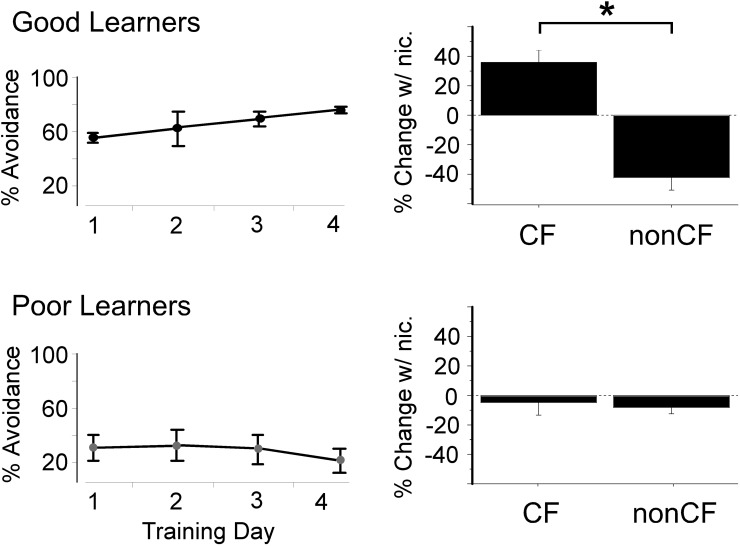
**“Good learning” in an auditory-cued task is associated with nicotine-induced enhanced response to CF stimuli and reduced response to spectrally distant (nonCF) stimuli in auditory cortex.** Left column depicts active avoidance behavior for “good” and “poor” performing groups (*n* = 4 each) across four days of training (50 trials per day). Y-axis indicates percent of trials during which a rat successfully avoided a shock after hearing a tone cue. Right column shows effect of systemic nicotine on tone-evoked local field response recorded in the middle layers of auditory cortex for the same animals after completion of all training. Response expressed as percent change from response after systemic saline, averaged across intensity for each animal (20–60 dB re. threshold). ^*^Paired *t*-test, *p* < 0.05. CF, characteristic frequency. Figure modified from Liang et al., 2008; reproduced with permission.

A surprising source of variability identified in recent studies is variability of nAChR expression levels or function among individuals. In a study from our laboratory, rats were first trained in an auditory-cued behavioral (active avoidance) task, and after four days of training were tested for effects of systemic nicotine on tone-evoked responses in auditory cortex (Liang et al., 2008). Receptive fields were probed at two points: at the characteristic frequency (CF, frequency with the lowest threshold) and at a second frequency 2–3 octaves below CF (referred to as “nonCF”). Averaged across all animals, systemic nicotine significantly enhanced responses to CF stimuli with little effect on the nonCF response, indicating a differential effect on the “center” and “edge” of the receptive field, respectively. However, when the physiological results were grouped according to the animals' ability to learn the task, a striking result emerged: in animals characterized as good learners, nicotine simultaneously enhanced responses to CF stimuli and reduced responses to nonCF stimuli (Figure 4). In contrast, for poor learners nicotine had little effect on acoustic responses. These results provided a first suggestion that nicotine sharpens sensory receptive fields, but only in animals that learn a sensory task well.

Other studies supported these initial findings. In a follow-up study, animals trained on the same auditory-cued task were subsequently imaged using PET and autoradiography methods to estimate the distribution and density of α4β2^*^ nAChRs in several forebrain regions (Bieszczad et al., 2012). Density of nAChRs in each region was correlated with measures of behavioral performance. Interestingly, nAChR density in the region of the auditory thalamocortical pathway (out of all auditory forebrain regions tested) was strongly correlated with performance in individual animals. While the presence of nAChRs in thalamocortical white matter may seem surprising, it also has been observed in human (Ding et al., 2004) and it is consistent with the recent demonstration that nicotine enhances the excitability of myelinated auditory thalamocortical axons (Kawai et al., 2007). The finding that individual variation in nAChR expression levels or function may relate to behavior is reminiscent of the genetic findings described above relating α4^*^ nAChRs and attentional performance in human subjects (Espeseth et al., 2010). Overall, these studies suggest that individual variability in nAChR expression may contribute to variability in sensory-cognitive performance.

## Nicotinic enhancement of response selectivity in primary auditory cortex

**Figure 5:**
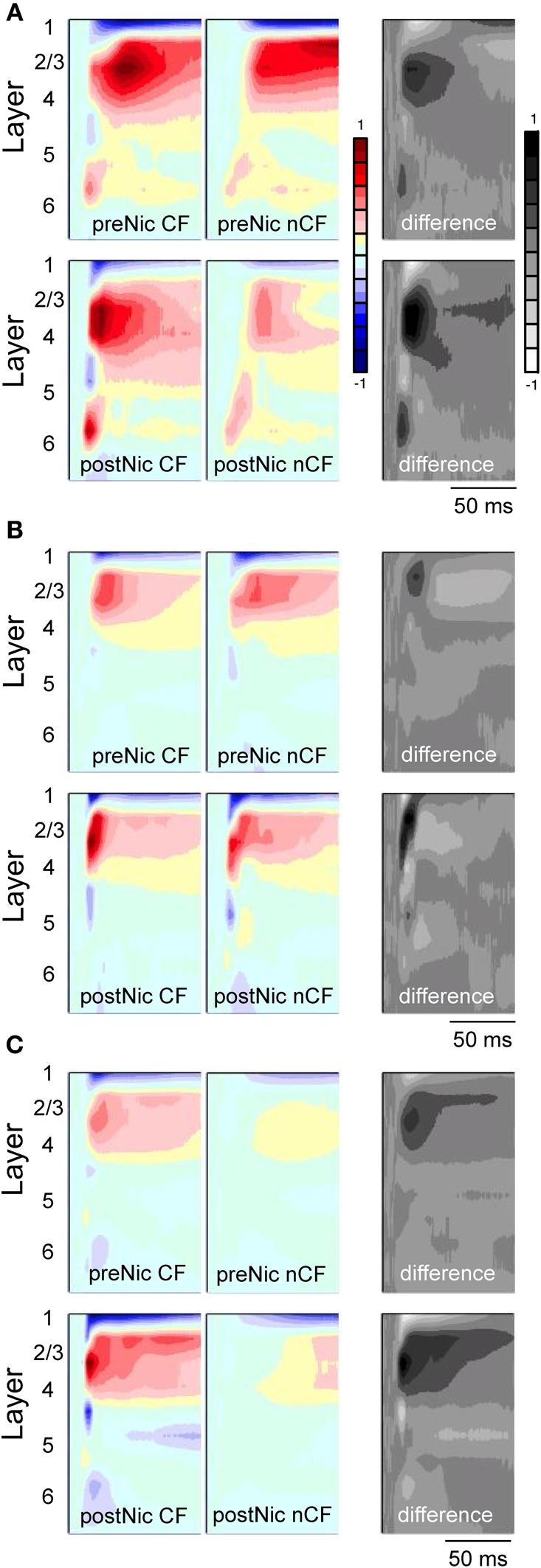
**Effects of systemic nicotine on tone-evoked CSD profiles in primary auditory cortex of three individual mice (A, B and C).** In each case, top row is control response and bottom row is post-nicotine (0.7 mg/kg, dose calculated as free base). CSD profiles show response to CF stimulus (left column) and a spectrally distant nonCF stimulus (nCF, middle). Difference profiles (right column) are obtained by subtracting nonCF-evoked CSD profiles from CF-evoked CSD profiles. CSD profiles normalized to maximum sink (reds) and source (blues) across all conditions for each animal; difference profiles normalized separately to maximum positive (black) and negative (white) differences. Tone onset at start of each record, duration 100 ms, intensity 65–70 dB SPL. Laminar depths of responses are estimated based on location of the earliest onset current sink, which was assigned to mid-layer 4. Data from Kawai et al., 2011 and Intskirveli and Metherate, 2012.

Given the potential importance of our initial finding that systemic nicotine may sharpen receptive fields in auditory cortex and thereby impact cognitive function (Figure 4) (Liang et al., 2008), we explored this finding in more detail in two subsequent studies (Kawai et al., 2011; Intskirveli and Metherate, 2012). Whereas the initial finding was based on microelectrode recordings from a fixed depth (approximately layer 4) in auditory cortex, the follow up studies used 16-channel multiprobes that spanned the entire cortical depth (100 μm separation between recording sites), in order to derive current-source density (CSD) laminar profiles for tone-evoked responses in mouse primary auditory cortex (Figure 5). As before, receptive fields were probed at CF and at a second frequency two octaves below CF (nonCF), but with significantly greater detail regarding the laminar and temporal flow of information through cortex. (In CSD analysis, current sinks—red colors in Figure 5—reflect presumed activation of excitatory synapses.) Tone-evoked CSD profiles were determined prior to, and after, systemic administration of nicotine.

**Figure 6:**
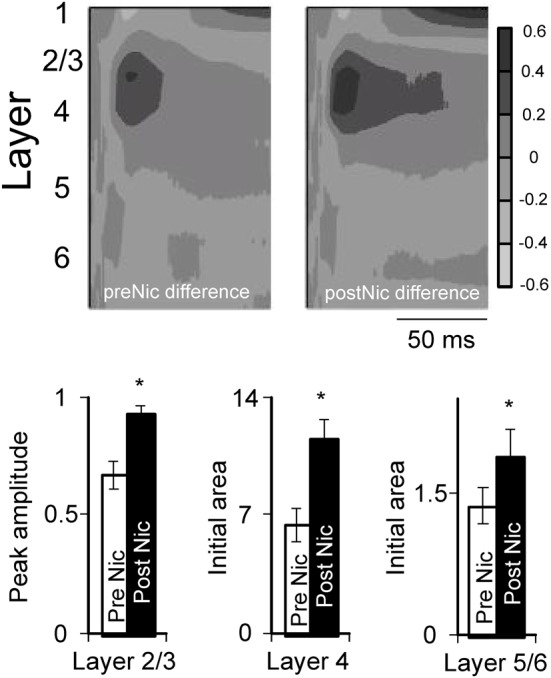
**Effect of systemic nicotine on group difference profiles.** Difference profiles normalized for individual animals (e.g., Figure 5) were averaged to create group profiles (*n* = 15 mice); these profiles do not represent actual sinks and sources, but instead reflect the contrast between CF-evoked responses and nonCF-evoked responses. Histograms show effect of nicotine on difference measures in layers 2/3, 4, and 5/6, using peak amplitude (layer 2/3) or initial area (initial 5 ms for layer 4, and 3 ms for layers 5/6 are measured, rather than peak, since initial response is thought to reflect thalamocortical input). Note that the layer 5/6 difference responses are not visible in the group profile, due to their small magnitude. ^*^paired *t*-test: layer 2/3, *p* = 0.0056; layer 4, *p* < 0.0001; layer 5/6, *p* = 0.0074 (*n* = 14–15). Data from Kawai et al., 2011 and Intskirveli and Metherate, 2012.

Example effects of nicotine on tone-evoked CSD profiles from three individual animals are in Figure 5, with group data in Figure 6 that combine results from the two studies (Kawai et al., 2011; Intskirveli and Metherate, 2012). The three examples have in common representative features of nicotine's effects, yet also illustrate other features that exhibited greater individual variation. In general, nicotine exerted similar effects on CF-evoked responses, increasing amplitudes and decreasing latencies for the main current sinks found approximately in layer 4 (defined by the shortest-latency, presumed thalamocortical input), layer 2/3 (the main, intracortical response) and to a lesser extent, layer 5/6 (infragranular sink). In contrast, nonCF-evoked responses were more variable, both in terms of the pre-nicotine CSD profile as well as effects of nicotine: in some cases, post-nicotine CSD profiles were reduced overall (Figure 5A), and in other cases nonCF responses were affected only weakly (Figure 5C shows mild suppression of shorter-latency current sinks and mild enhancement of longer latency responses). Still other cases exhibited a mix of effects (Figure 5B shows enhancement of shorter latency nonCF-evoked response and lesser effects at longer latencies). Nevertheless, in all cases nicotine's effects on nonCF responses contrasted with its enhancement of CF-evoked responses, and overall suppression of nonCF-evoked profiles emerged in group data, as reported (Kawai et al., 2011; Intskirveli and Metherate, 2012), although the group data mask the diversity of effects.

Figure 5 also depicts, for each animal, a “difference” profile in each condition obtained by subtracting layer-by-layer the nonCF-evoked response from the CF-evoked response. The difference profile does not indicate the location of actual current sinks and sources (hence the alternate, gray scale), but instead indicates the contrast between CF- and nonCF-evoked responses. The effect of nicotine on difference profiles is consistent across animals—despite its variable effect on nonCF responses nicotine enhances difference profiles, increasing amplitudes and decreasing latencies especially in layers 2/3 and 4. Thus, regardless of nicotine's effect on nonCF-evoked responses, nicotine consistently increases the contrast between CF- and nonCF-evoked responses. Group data for difference profiles across all animals in both studies are shown in Figure 6, which indicates that nicotine enhances the favoring of CF-evoked responses for each current sink in layers 2/3, 4, and 5/6.

## Conclusion

Research on nAChRs and their functions in the brain has progressed to where it is useful to begin to link cellular mechanisms to behavioral consequences. Nicotinic enhancement of sensory-cognitive function likely involves nAChRs located within primary sensory cortex, where modulation of receptive fields could significantly affect downstream information processing in higher cortical areas. The model proposed here—that attentional filtering may involve activation of nAChRs within sensory cortex to narrow receptive fields and enhance responsiveness within the narrowed receptive fields—must be considered speculative, but it represents an empirical step towards a mechanistic understanding of cognitive functions, and towards the development of selective nicotinic agents for treatment of specific cognitive dysfunctions. This mechanism is likely only one of multiple mechanisms underlying attention-dependent regulation of receptive fields, since a variety of receptive field changes may occur—e.g., increased tuning width, decreased tuning width, and complex changes in tuning (Fritz et al., 2003; Seriès et al., 2004; Reynolds and Heeger, 2009). Immediate future goals are to understand mechanisms of sensory receptive field modulation by nAChRs, with the longer-term goals of relating these findings to other (including non-sensory) cortical regions and to perceptual consequences. Although much work remains, the pace of progress is rapid and these goals, while ambitious, are within reach.

### Conflict of interest statement

The authors declare that the research was conducted in the absence of any commercial or financial relationships that could be construed as a potential conflict of interest.
